# Gender Differences in the Impact of COVID-19 Lockdown on Potentially Addictive Behaviors: An Emotion-Mediated Analysis

**DOI:** 10.3389/fpsyg.2021.703897

**Published:** 2021-11-15

**Authors:** Giuseppe Attanasi, Anna Maffioletti, Tatyana Shalukhina, Coralie Bel, Faredj Cherikh

**Affiliations:** ^1^GREDEG, CNRS, University of Côte d’Azur, Nice, France; ^2^ESOMAS, University of Turin, Turin, Italy; ^3^Addictology Unit, University Hospital of Nice, Nice, France

**Keywords:** COVID-19, lockdown, non-creative activity, addiction, food, smartphone, videogames, negative emotions

## Abstract

We study the impact of the spring 2020 lockdown in France on gender-related potentially addictive behaviors and associated negative emotions. We rely on an online survey we administered 1 week after the beginning of the lockdown, with responses collected within 2 weeks after the beginning of the lockdown (*N* = 1,087). We focus on potential addictions to non-creative activities as food consumption and smartphone usage (female-related), and videogame play (male-related). We find that women were about 1.6 times more likely than men to losing control of their usual diet and about 2.3 times more likely than men to increase smartphone usage, while no significant gender effect is detected as for increased videogame play. This is since the negative emotions driving the increase of female-related non-creative activities (sadness, discouragement, and nervousness) were themselves female-related, while the negative emotions driving the increase of male-related non-creative activities (boredom, emptiness, and stress) were shared by women too. Our study supports the intuition that the same negative emotion induced by COVID-19 side-effects could lead to different potentially addictive behaviors; this difference is explained by the interplay between different gender’s sensitivities to such emotion and different gender’s preferences for specific non-creative activities.

## Introduction

As almost 1 year has passed from the moment WHO classified and declared COVID-19 as a “global pandemic,” the battle against this virus is still ongoing. And even if society is getting close to the resolution of the reason causing the virus spread, implementing a vaccine campaign for adults while still finding the proper treatment for the illness, the psychological, sociological, and economic negative effects of the pandemic and the subsequent series of lockdown periods throughout the world will, unfortunately, be long-lasting. In this paper, we focus on the former effects, i.e., individuals’ psychological attitudes and behavioral responses to the unforeseen contingencies that the spread of COVID-19 has brought with it.

In particular, the lockdown has triggered potential addictions to unhealthy behaviors including increased smoking (Jackson et al., 2021), alcohol consumption (Cummings et al., 2021), and has also been associated with changes in eating habits with a sharp increase in binge and compulsory eating and overall weight gain (Cherikh et al., 2020). Indeed, eating is a simple way to control stress and anxiety and it has been predicted that the prevalence of obesity would increase sharply due to the pandemic and the related lockdown periods (see Alifano et al., 2020, and follow-up papers).

This paper presents the analysis of the lockdown impact on developing potentially addictive behaviors concerning gender. It relies on the data generated by an online survey administered in France by two of the paper authors, belonging to the Addiction Unit of the Department of Psychiatry of University Hospital of Nice, 1--3 weeks after the beginning of the spring 2020 lockdown. As for women, 1 week after the beginning of the lockdown in France, law-enforcement interventions following reports of domestic violence have increased by 32%,^1^ with similar tendencies being detected in other European countries during the same timeframe.^2^

France was the first European country that confirmed a COVID-19 case on the 24th of January. Due to the increasing number of cases, the first 2-week lockdown was implemented starting from 17th of March 2020, which was then extended till the 10th of May 2020. During this period, the most affected areas of France were Paris (Île-de-France) and the Northeast (Souty et al., 2021). The main restrictions imposed by the government’s decrees for the lockdown period included the following (Renou, 2020): only essential services (food shops, pharmacies, banks, newsagents, and petrol/service stations) and all essential public services were authorized to remain open; closure of schools and universities; ban on religious gatherings with some exceptions like funeral ceremonies; ban on traveling with some exceptions due to professional, family, or health-related issues.

Our survey was conducted during the 2nd week of the spring lockdown of 2020 in France, namely between the 24th of March and the 31st of March 2020. A stable panel (*N* = 1,087) of adult subjects was surveyed during 7 consecutive days, i.e., after 1–2 weeks from the beginning of the lockdown. The dataset contains information about people’s emotional states and reactions to these states in the context of potentially developing addictions to food consumption, smartphone usage, and videogame play. The main reason for our interest in these three types of addictive behavior during the 2020 lockdown period relies on previous psychological literature suggesting gender differences in non-creative activities leading to these addictions through time.

As for *food consumption*, studies in psychology have detected an association between the recurrent occurrence of binge eating and behavioral manifestations of loss of control over eating behavior on the one side and marked distress due to psychological seizures on the other side (Davis, 2013). The psychological suffering associated with these attacks is a criterion for the diagnosis of binge eating disorder. These attacks are also most often triggered by dysphoric states such as anxiety and depression. In this regard, food addiction is not a separate entity from binge eating, but rather a severe subtype of it, strongly marked by the impulsive component. A meta-analysis of YFAS food addiction studies in 2014 found a 20% prevalence of food addiction in the adult population tested (Pursey et al., 2014). The prevalence of it was twice as high in overweight people. Food addiction was also more prevalent in adults over 35 and – what is interesting for the scope of our study – women were more affected than men.

As for the other two addiction items that we included as items in our survey, consistent findings are lacking on whether the risks of developing those addictions vary across genders (see, e.g., the survey in Chen C. et al., 2017). However, when disentangling specific behaviors, gender differences emerge. Toda et al. (2006) found a higher potential for females to become addicted to online services. For example, in the context of *smartphones*, research indicates that the risk of addiction is higher for females (Walsh et al., 2011; Hong et al., 2012). Conversely, males have been detected to be more likely to be addicted to *videogames* than females (Bianchi and Phillips, 2005; Wittek et al., 2016), with young adult males being at the greatest risk for videogames (King et al., 2012).

In our study, we focus on the psychological determinants of the increase in these three non-creative activities (i.e., potentially addictive behaviors) because of the COVID-19 lockdown, and look for gender effects, assuming – in line with the above-mentioned psychological literature – that food consumption and smartphone usage are non-creative activities to which women are more prone than men (i.e., female-oriented), and that videogame play is a non-creative activity to which men are more prone than women (i.e., male-oriented).

Several studies have tried to identify the main psychological drivers for the three aforementioned potentially addictive behaviors (see section “Research Hypotheses”). However, research on gender differences on these psychological determinants remains scarce. Our study aims at filling this gap by analyzing the increase in these non-creative activities due to the COVID-19 lockdown, detecting possible correlations between gender-related potentially addictive behaviors and associated negative emotions. As for the latter, we consider several negative emotional states – sadness, discouragement, nervousness, boredom, emptiness, and stress –, relating them to the COVID-19 lockdown and the increase in non-creative activities run at home because of this lockdown. Our results support the intuition that the same negative emotion induced by COVID-19 side-effects could lead to different potentially addictive behaviors; this difference is explained by the interplay between different gender’s sensitivities to such emotion and different gender’s preferences for specific non-creative activities.

The remaining part of this article is structured as follows. Section “Research Hypotheses” introduces our research hypotheses, relying on the previous literature on the psychology of addiction and emotions. Section “Methodology” presents the data and the methodology followed. Section “Results” presents the main results in light of the hypotheses introduced in the section “Research Hypotheses.” Section “Discussion and Conclusion” discusses the results and concludes.

## Research Hypotheses

In this section, we elaborate on the hypotheses we want to test about the impact of the COVID-19 lockdown on potentially addictive behaviors of women and men, by looking at the driving (negative) emotions of this impact. We are interested in potential addictions to food consumption, smartphone usage, and videogame play.

The phenomenon of *addiction* is commonly known in modern society. Yet, since it deals with a wide range of behaviors, there are many ways to characterize it.^3^ The definition we apply in our study is suggested in a publication on models of addiction prepared by the European Monitoring Centre for Drugs and Drug Addiction (EMCDDA), where addiction is defined as “a repeated powerful motivation to engage in a purposeful behavior that has no survival value, acquired as a result of engaging in that behavior, with significant potential for unintended harm” (West, 2013). Here the emphasis can be put on the word “harm,” since any behavior can be seen as potentially addictive, e.g., gardening. However, to justify academic or medical usage of this term, the feature of negative consequences must be present (Griffiths, 2005).

There are two main types of addictions: substance addiction and non-substance or *behavioral addiction*. The latter is a relatively new concept, as only in 2010 this term was included in the Diagnostic and Statistical Manual of Mental Disorders (DSM-5) as the official classification of psychiatric diagnoses (Derevensky et al., 2019). Several behaviors can be referred to as potentially addictive. For example, gambling (Griffiths, 1995), sex (Carnes, 2001), exercise (Terry et al., 2004), eating (Pelchat, 2009), overeating (Orford, 2001), Internet usage (Griffiths, 2000), and videogame play (Griffiths, 2002). There are certain distinctions between behavioral addiction and traditional substance addiction in terms of their development, effects, and treatment. However, the way they can be generally defined is remarkably similar. Behavioral addiction shares “the most evident characteristic of addiction, i.e., continuous substance intake (addictive behavior) despite negative consequences, which is associated with craving and lack of control” (Albrecht et al., 2007).

As anticipated in the Introduction, ours is not a study of substance addiction driven by the lockdown. We rather focus on non-substance addictions, namely potential addictions to non-creative activities as food, smartphone, and videogames. The reason for this focus is due to the nature itself of the COVID-19 lockdown: due to the prolonged confinement at home, behavioral addictions to everyday objects and needs would suddenly emerge. And given that our survey was carried out during the 2nd week of the first COVID-19 lockdown in France, we thought that the first potentially addictive behaviors emerging would not concern drugs or medicines but rather more “familiar” habits, which home confinement would have easily offered.

More precisely, relying on Scitovsky (1992) psychological notion of ‘‘*comfort*’’ as opposed to the one of ‘‘novelty,’’^4^ we define “comfort” as setting arousal at its optimum level, i.e., comfort implies “behavior that satisfies various bodily and mental needs and so lowers arousal that is too high; it also includes behavior which combats boredom and so raises arousal that is too low.” Comforts can be viewed as products that relieve pain, “fatigue, eliminate bother and save time” (Scitovsky, 1992: 59–61, 112–113). Sticking to the habits is another type of comfort. An immediate reward (reduction in arousal) followed by any action reinforces this action and consequently forms a habit. Once the habit is formed one continues to follow it not so much for the rewarding effect of the action itself, but mainly for avoiding the discomfort associated with interrupting it. This cycle resembles the one related to harmful addictions. Scitovsky sees harmful addictions as one of the types of habits (Scitovsky, 1992).

The bias for comfort and against novelty appears to be due to the lack of training in the skill of consumption. Eating, interacting with a smartphone, playing videogames are all activities that do not require special skills, as compared to e.g., reading a book, which is a more creative activity.^5^ A bigger variety of consumption skills provides a consumer with a wider range of sources of stimulation. In other words, a skilled consumer is capable to enjoy more creative activities, due to his/her intrinsic motivation (Attanasi et al., 2021). Failure to experience pleasure from creative activities can explain why people are engaging in harmful and addictive ones, which also give them certain stimulation without any required skills for that (Scitovsky, 1992).

The association between activities that do not require special skills, i.e., non-creative activities, and the formation of habits during the COVID-19 lockdown leads to our first research hypothesis, which relies on the intuition that, due to the huge amount of time spent at home during the 1st weeks of the lockdown, people have looked for comfort by enhancing the risk of potential addictions to non-creative activities.

***Hypothesis 1 [Non-creative activities]:*** *COVID-19 lockdown has enhanced non-creative activities (food consumption, smartphone usage, and videogame play).*

The second research hypothesis comes from the observation that the three non-creative activities on which our study focuses – food, smartphone, and videogames –, are at the base of specific potential addictions that usually vary across genders, as the psychological literature discussed in the Introduction highlights. Relying again on Scitovsky’s (1992) link between a search for comfort and addiction to non-creative activities, it seems natural to take on each gender “specializing” during the lockdown on their more frequent non-creative activity before the lockdown. More precisely, we hypothesize that the abrupt COVID-19 lockdown has led people to relieve pain and find comfort by sticking to their habits, thereby boosting gender differences in non-creative activities, with women increasing more than men food and smartphone consumption – which were already female-based activities –, and man increasing more than women videogame play – which was already a male-based activity.

***Hypothesis 2 [Gender difference in non-creative activities]:*** *COVID-19 lockdown has enhanced more gender-related non-creative activities, with females (resp., males) increasing food and smartphone (resp., videogames) consumption more than male (resp., female).*

Our third research hypothesis focuses on the emotional drivers of the three aforementioned potentially addictive behaviors. Our main theoretical reference is Loewenstein (1999), who sees “visceral factors” (drive states such as hunger, thirst, sexual desire, emotions, etc.) playing the leading role in determining behavior. Therefore, emotions are a main defining feature of potentially addictive behavior, as the psychological literature has shown for eating loss of control, smartphone usage increase, videogame play increase.

As for food consumption, research in emotional drivers of eating is plentiful (see Canetti et al., 2002 for a survey). Mehrabian (1980) found that higher food consumption was reported during boredom, depression, and fatigue, and lower food intake was reported during fear, tension, and pain. Lyman (1982) showed a greater tendency to consume healthy food during positive emotions and a greater tendency to consume junk food during negative emotions. Patel and Schlundt (2001) found that meals eaten in positive and negative moods were significantly larger than meals eaten in a neutral mood. Macht (1999) detected higher levels of hunger during anger and joy than during fear and sadness, with anger linked to an increase of impulsive eating and joy linked to an increase of hedonic eating.

As for smartphone usage, Chen B. et al. (2017) identified associations between smartphone usage, psycho-behavioral factors, and smartphone addiction, and showed that the associations differ between males and females. Mediation of specific negative emotions in smartphone addiction has been documented. In particular, a plethora of studies reports a strong boredom-smartphone interaction (e.g., Matic et al., 2015; Elhai et al., 2018; Leung, 2020) and an equally strong stress-smartphone interaction (e.g., Wang et al., 2015; Samaha and Hawi, 2016; Vahedi and Saiphoo, 2018), and emptiness has been detected as mediating the association between pathological narcissism and problematic smartphone use (Zerach, 2021).

As for videogame play, recent psychological studies have found associations with both positive mental states (e.g., Villani et al., 2018) and negative mental states (e.g., Gibbons and Bouldin, 2019). Among the latter studies, Loton et al. (2016) have documented a significant relationship between videogame addiction and symptoms of depression, anxiety, and stress.

With all the above, we elaborate our third research hypothesis on the effect of overcoming negative emotions in undertaking potentially addictive behaviors, separately for each non-creative activity – food, smartphone, and videogames – and for each negative emotion included in our survey – sadness, discouragement, nervousness, boredom, emptiness and stress.

***Hypothesis 3 [Emotional mediation in non-creative activities]***: *Potentially addictive behaviors are more likely to occur for subjects with negative emotions, and especially for those who tend to overcome them with the help of non-creative activities.*

Our last hypothesis starts from the consideration that the COVID-19 pandemic has caused significant effects on our mental health and that these effects have been different for different genders and different gender-related emotions. In this regard, Guadagni et al. (2020) report that the COVID-19 pandemic in Canada has affected women differently than it has affected men: women reported more sleep troubles, more symptoms of anxiety and depression, and greater empathy for others. In the same vein, García-Fernández et al. (2021) assess that during the 1st weeks of the COVID-19 lockdown in Spain, women presented greater severity in symptoms of anxiety, depression, and acute stress. Ausín et al. (2021) confirm gender-related differences in the psychological impact of confinement in Spain, with COVID-19 having a greater psychological impact on women than men.

Other studies provide support to gender differences in the increase of negative emotions due to COVID-19 sudden health and lifestyle changes (Ahuja et al., 2020; Galasso et al., 2020; García-Fernández et al., 2021; Shockley et al., 2020; Bernabe-Valero et al., 2021; Kidd et al., 2021), although they do not provide a final say on which specific emotion women are more affected than men, and whether the gender difference is driven by that specific emotion or by the fixed effect of a mental state degraded by COVID-19 side-effects. Furthermore, like those of Guadagni et al. (2020) and Ausín et al. (2021), these studies focus on at least one of the emotions we included in our survey, although none of them analyze gender effects on sadness, discouragement, nervousness, boredom, emptiness, and stress within the same survey. With this, we formulate our last hypothesis.

***Hypothesis 4 [Gender differences in emotional mediation]***: *The negative emotions driving gender-related potentially addictive behaviors are themselves gender-related.*

This last hypothesis aims at detecting whether women’s (resp., men’s) specific potentially addictive behavior is correlated with specific female-related (resp., male-related) negative mental states due to the COVID-19 lockdown, or whether gender-related specific potentially addictive behavior is independent of gender-related emotions.

The next section describes how the survey was designed and implemented, to obtain the data used to test our four research hypotheses.

## Methodology

Our survey was conducted during the 2nd week of the spring lockdown of 2020 in France, namely between March 24th and March 31st, 2020. The questionnaire was designed by two of the paper authors within the Addictology Unit of University Hospital of Nice during the 1st week of the lockdown. More precisely, the base of the questionnaire was represented by the same questionnaire that patients of the Addictology Unit of the Archet Hospital in Nice are asked to fill in since 2016, i.e., since Faredj Cherikh, one of the paper authors, is Head of this Unit. That questionnaire is meant to screen for addictive behaviors during everyday life. This is the first time that this questionnaire has been administered to a general population. Some of the questionnaire items were adapted in order to detect lockdown side-effects. Other questions related to lockdown side effects were added.

The questionnaire was distributed through social media in France during the 2nd week of the lockdown. More precisely, the online survey was mainly shared *via* Facebook, since at the time of the survey it was the social media with the highest market share in France (58.7%).^6^ The survey, conducted in French and taking less than 10 min on average, was open to any adult person undergoing lockdown in France. The population of the study was unspecific. 1,087 individuals replied to the questionnaires within the first 7 days of online sharing of the questionnaire.

The questionnaire contained questions about experiencing various emotional states and performing different potentially addictive behaviors. The questionnaire on which the survey relied was made of four families of items: *socio-demographics*, *emotional states*, *non-creative activities*, and *potentially addictive behaviors*. For emotional states, our questionnaire includes two types of items: emotions elicited without relating them to specific behavior (*unconditional*: sadness, discouragement, and nervousness) and emotions elicited conditionally to the (non-creative) activity – web, food, and videogame consumption – aimed at managing them (*conditional*: boredom, emptiness, and stress).

Table 1 presents summary statistics of the selected variables, while the detailed description of these variables can be found in Supplementary Appendix Table A1. Supplementary Appendix B reports the English translation of the questionnaire.^7^

**TABLE 1:** Summary statistics of selected variables.

	**Obs.**	**Type**	**Mean**	**SD**	**Min**	**Max**

**Socio-demographics**						
Female	1.087	D	0.75	0.44	0	1
Age 1 (18–35)	1.087	D	0.64	0.48	0	1
Age 2 (36–55)	1.087	D	0.28	0.45	0	1
Age 3 (56–75)	1.087	D	0.07	0.26	0	1
Age 4 (>75)	1.087	D	0.01	0.06	0	1
Health 0 (good)	1.087	D	0.77	0.42	0	1
Health – 1 (pathologies)	1.087	D	–0.21	0.41	0	1
Health – 2 (serious pathologies)	1.087	D	–0.02	0.13	0	1
Lockdown period 1 (<1 week)	1.085	D	0.11	0.31	0	1
Lockdown period 2 (1–2 weeks)	1.085	D	0.81	0.39	0	1
Lockdown period 3 (2–3 weeks)	1.085	D	0.08	0.28	0	1
Employed	1.085	D	0.48	0.50	0	1
Stay at home	1.085	D	0.75	0.43	0	1
In relationship	1.087	D	0.79	0.41	0	1
Children	1.086	D	0.37	0.48	0	1
Physical activity 0	1.086	D	0.31	0.46	0	1
Physical activity 1 (infrequent)	1.086	D	0.42	0.49	0	1
Physical activity 2 (frequent)	1.086	D	0.27	0.45	0	1

**Emotional states**						

Boredom food	1.086	D	0.43	0.50	0	1
Boredom web	1.086	D	0.79	0.40	0	1
Boredom videogame	1.086	D	0.30	0.46	0	1
Emptiness food	1.086	D	0.37	0.48	0	1
Emptiness web	1.086	D	0.66	0.47	0	1
Emptiness videogame	1.086	D	0.24	0.43	0	1
Stress food	1.084	D	0.37	0.48	0	1
Stress web	1.085	D	0.45	0.50	0	1
Stress videogame	1.085	D	0.19	0.39	0	1
Sadness	1.082	D	0.22	0.42	0	1
Discouragement	1.082	D	0.37	0.48	0	1
Nervousness	1.079	D	0.45	0.50	0	1

**Non-creative activities**						

Smartphone sms-calls	1.078	D	0.88	0.32	0	1
Smartphone soc-network	1.078	D	0.89	0.31	0	1
Smartphone games	1.078	D	0.41	0.49	0	1
Videogame play	1.083	D	0.45	0.50	0	1

**Potentially addictive behaviors**						

Eating loss of control	1.085	D	0.26	0.44	0	1
Smartphone usage increase	1.084	D	0.88	0.32	0	1
Videogame play increase	1.083		1.18	1.35	0	3

As for subjects’ idiosyncratic features, the sample is gender skewed: 74.7% of respondents were female. Although female subjects in the survey period were majoritarian both in the French adult population^8^ and in the French Facebook adult users^9^ during the period of our survey, this does not fully explain the fact that three out of four subjects of our survey respondents were female. The fact that women are more likely to participate in surveys than men is well documented in the literature (see Curtin et al., 2000; Moore and Tarnai, 2002, and Singer et al., 2000 for traditional modes of survey administration, and Smith, 2008 for online surveys). Our guess is that, when receiving our survey invitation for evaluating the impact of the lockdown on the state of psychological health, females participated more than males due to their worse psychological health during the lockdown – which we will document in the section “Results” –, which implied a higher willingness to report it. However, given that the fraction of male responders was high enough (275 subjects), we are confident that results of non-parametric tests of gender differences and odds ratios of gender dummy variables in the regression models in the section “Results” should not depend on the high number of female respondents in our sample.

As for age, the prevailing age ranges correspond to younger generations: 64.49% of our respondents belonged to the 18--35 age group (dummy ‘‘Age 1’’), 27.87% to the 36--55 age group (dummy ‘‘Age 2’’), 7.27% to the 56--75 age group (dummy ‘‘Age 3’’), and the remaining 0.37% were older than 75 (dummy ‘‘Age 4’’). In the data analysis of section ‘‘Results’’ we will pool Age 3 and Age 4 in a single dummy Age 3--4 because of the negligible number of subjects in the latter category. We acknowledge that our sample is not representative of the wider French population as for age distribution.^10^ However, our age distribution is quite representative of social media users in France:^11^ the significantly higher share of young respondents in our sample is in line with studies showing that younger subjects are more willing to participate in app-based surveys (see, e.g., Mulder and de Bruijne, 2019).

As for the remaining socio-demographic variables, most respondents reported being in a relationship (79%) and being in a good health condition with no pathology (77%, dummy “Health 0”) at the beginning of the lockdown. As expected, given the lockdown restrictions, subjects in our sample mainly stayed at home during the 1st 2 weeks of the lockdown, except for those who had a job outside (25%). The sample is balanced as far as the employment rate is concerned: 48% of people had a job. Some of them were working at home while others were still working outside homes during the lockdown. Physical activity during the lockdown was smoothly distributed across no activity (31%, dummy “Physical activity 0”), infrequent activity (42%, dummy “Physical activity 0”), and frequent activity (27%, dummy “Physical activity 2”). The modal lockdown period was between 1 and 2 weeks (dummy “Lockdown period 2”).

As far as emotional state variables are concerned, the questionnaire includes two types of items. First, three items that elicit experiencing given emotions – sadness, discouragement, and nervousness – without relating them to specific addictive behavior. Second, three blocks of symmetric items, each of them eliciting the way the respondent managed three other emotions – boredom, emptiness, and stress – through specific non-creative activities. The three blocks refer, respectively to food, internet, and videogame consumption.

The questionnaire also includes three items detailing smartphone dependence, disentangling it among (i) SMS and calls, (ii) social networks, and (iii) games, identified as the main reasons for smartphone usage during the lockdown. These variables capture behavioral levels during the lockdown. The remaining items of addictive behavior linked to alcohol and medicine consumption are not analyzed in our study, since a negligible number of respondents (less than 7% on average over all these items) indicated alcohol or medicine consumption. As anticipated in the Introduction, this was expected, since our survey was run after only 1--2 weeks from the beginning of the lockdown, hence new addiction to alcohol and/or medicines due to the lockdown was unlikely, apart from those subjects already being addicted to alcohol and/or medicine consumption before the lockdown. Our study focuses instead on lighter forms of potential addictions that characterize a ‘‘normal’’ lifestyle -- food, smartphones, and videogames. Emerging after less than 1 month of lockdown, these behaviors might become dangerous in the medium-long term -- e.g., obesity, muscle pain, social isolation, mood swings, and decreased ideation. These ‘‘potentially addictive behaviors’’ variables capture changes in behavior during the lockdown as compared to the pre-lockdown levels. More precisely, for food consumption we focus on the dummy variable ‘‘Eating loss of control,’’ and for smartphone consumption, we focus on the dummy variable ‘‘Smartphone usage increase,’’ both stated with respect to the pre-lockdown period. For videogame consumption, we focus on the categorical variable ‘‘Videogame play increase,’’ with 0 indicating no activity, 1 indicating decreased activity, 2 indicating stable activity, and 3 indicating increased activity with respect to the pre-lockdown period. Here we assume that subjects declaring that they were not playing videogames during the lockdown were not playing them either before the lockdown (otherwise, they would have indicated decreased or stable activity). This is consistent with statistics on the wider French population: around 45% of our sample reported no videogame activity compared to 48% of French not playing videogames regularly and 39% of them not playing videogames at all.^12^

## Results

Summary statistics reported in the last section of Table 1 (“Potentially addictive behaviors”) provide **strong support for Hypothesis 1** as for the significant increase in non-creative activities during the 1st week of COVID-19 lockdown. In fact, after only a few weeks of lockdown: 26% of our sample felt like losing control of their usual diet; 88% of the sample started to use the smartphone more respect to the pre-lockdown period; 64% of those playing videogames reported increased activity respect to the pre-lockdown period, this fraction being significantly higher than the one of those reporting a stable activity (33%) or a decreased activity (3%) consider together, according to a Chi-square test of differences in proportions (*p*-value < 0.001).

The central section of Table 1 provides **first support for Hypothesis 3** on increase in non-creative activities being driven by specific negative emotions. Focusing only on boredom, emptiness, and stress, the weight of these three negative emotions seems to be similar for web, food, and videogame consumption, with *boredom*, always being the modal negative emotion that each of the three above-mentioned non-creative activities aims at mitigating. However, while for web and videogame consumption the relative weight of boredom (resp., 41 and 41%) is significantly higher than the one of emptiness (resp., 35 and 33%) and the one of stress (resp., 24 and 26%), for food consumption the three negative emotions have similar relative weights (36% boredom, 32% emptiness, 32% stress). Furthermore, inverting the direction of the analysis by moving from specific emotions to specific non-creative activities, the fraction of respondents managing boredom with web (79%) is significantly higher than the one of those managing boredom with food (43%) or videogame (30%) consumption (Kruskal–Wallis test of differences in distributions, *p*-value < 0.001). A similar result is found for emptiness and stress, with the fraction of those who managed it with web (resp., 66 and 45%) being significantly higher than the one of those managing it with food (resp., 47 and 37%) or videogame (resp., 24 and 19%) consumption (Kruskal–Wallis test of differences in distributions, *p*-value < 0.001). Therefore, boredom seems to be the leading negative emotion for non-creative activities during the 1st weeks of lockdown. However, the role of other negative emotions like sadness, discouragement, and nervousness cannot be assessed by looking at the statistics in Table 1. The regression analysis in the sections “Eating Loss of Control,” “Smartphone Usage Increase,” and “Videogame Play Increase” will help clarify the relative weight of these other three emotions on the lockdown-related increase in the three non-creative activities we are interested in in this study.

Table 2 reports the results of Mann–Whitney tests on the difference in the medians of the distributions of each variable of Table 1, disentangling by female vs. male. Variables for which there is a significant gender difference in favor of the female (resp., male) side are in bold (resp., Italic) fonts.

**TABLE 2:** Gender differences in idiosyncratic features, emotional states, and behavior.

	**Female**	**Male**	***p*-value**

**Socio-demographics**			
**Age (1 to 4)**	1.46 (0.23)	1.36 (0.35)	0.04**
Health (−2 to 0)	−0.25 (0.02)	−0.25 (0.03)	0.90
Lockdown period (1 to 3)	0.97 (0.02)	0.99 (0.03)	0.50
Employed	0.48 (0.02)	0.47 (0.03)	0.77
Stay at home	0.75 (0.02)	0.77 (0.03)	0.43
**Living with partner**	0.81 (0.02)	0.72 (0.03)	0.00***
**Living with children**	0.40 (0.02)	0.28 (0.03)	0.00***
Physical activity (0 to 2)	0.97 (0.03)	0.92 (0.05)	0.36

**Emotional states**			

Boredom food	0.43 (0.02)	0.43 (0.03)	0.92
*Boredom web*	0.78 (0.01)	0.84 (0.02)	0.03**
*Boredom videogames*	0.22 (0.01)	0.55 (0.03)	0.00***
**Emptiness food**	0.38 (0.02)	0.32 (0.03)	0.09*
Emptiness web	0.66 (0.02)	0.68 (0.03)	0.57
*Emptiness videogames*	0.16 (0.01)	0.47 (0.03)	0.00***
**Stress food**	0.39 (0.02)	0.32 (0.03)	0.03**
Stress web	0.44 (0.02)	0.47 (0.03)	0.31
*Stress videogames*	0.13 (0.01)	0.37 (0.03)	0.00***
**Sadness**	0.24 (0.02)	0.18 (0.03)	0.03**
**Discouragement**	0.40 (0.02)	0.28 (0.03)	0.00***
**Nervousness**	0.49 (0.02)	0.32 (0.03)	0.00***

**Non-creative activities**			

**Smartphone smscalls**	0.90 (0.01)	0.81 (0.02)	0.00***
Smartphone socnetwork	0.89 (0.01)	0.90 (0.02)	0.73
Smartphone games	0.39 (0.02)	0.44 (0.03)	0.11
*Videogame play*	0.37 (0.02)	0.71 (0.03)	0.00***

**Potentially addictive behaviors**			

**Eating loss of control**	0.29 (0.02)	0.19 (0.02)	0.00***
**Smartphone usage increase**	0.90 (0.01)	0.83 (0.02)	0.00***
*Videogame play increase*	1.89 (0.08)	0.94 (0.05)	0.00***

Results of Mann–Whitney test; *p-value < 0.1, **p-value < 0.05, and ***p-value < 0.01.

As for socio-demographic variables, we see that in our sample female respondents are significantly older than male ones. Furthermore, significantly more female than male respondents are at home with their partner and/or their children. In the test of Hypotheses 1–4, we will check that none of these three socio-demographic variables (age, living with a partner, or with children) have a significant impact on potentially addictive behaviors, to assess that our results are not driven by socio-demographic sample bias.

The gender differences systematically detected as for emotional states variables in the central section of Table 2 provide **first support for Hypothesis 4**. Women show significantly higher sensitivity than men to unconditional emotions sadness (at the 5% level), discouragement, and nervousness (at the 1% level). Therefore, when sensitivity to negative emotions is elicited without relating them to specific addictive behavior – namely, sadness, discouragement, and nervousness – women disclose a higher sensitivity, thereby showing a more problematic psychological condition during the 1st week of the lockdown. Conversely, men show significantly higher sensitivity than women to boredom, emptiness, and stress, on average over the three non-creative activities they are aimed at managing (61 vs. 48% for boredom, 49 vs. 40% for emptiness, and 39 vs. 32% for stress, all differences being significant at the 1% level according to a Chi-square test of differences in proportions). Furthermore, disentangling these three negative emotions by the non-creative activity they are aimed at managing, men are more sensitive to all of them when they are related to videogame consumption, while women are more sensitive to two out of these three emotions (emptiness and stress) when related to food consumption. We interpret this as the first proof that the same negative emotion could lead to different potentially addictive behaviors; this difference is explained by the interplay between different gender’s sensitivities to these emotions and preferences for non-creative activities.

Finally, looking at the last section of Table 2, gender differences detected in the “potentially addictive behaviors” variables provide **first support for Hypothesis 2**. As predicted, women disclose a higher loss of control of food consumption and a higher increase of smartphone usage. Conversely, and again in line with Hypothesis 2, we detect a significantly higher median in males’ responses to the “Videogame activity increase” question (0 = no; 1 = decreased; 2 = stable; and 3 = increased activity), suggesting a higher increase in videogame play during the lockdown for males than for females. However, as we will see in the section “Videogame Play Increase,” the latter result is driven by the fixed effect of males playing videogames more than females regardless of the lockdown.

In the next three subsections, we separately test Hypotheses 2 and 3 on each of the three potentially addictive behaviors. More precisely, we present and discuss regression results starting from the problem of overeating (Section “Eating Loss of Control”) and then analyzing smartphone dependence (Section “Smartphone Usage Increase”); the final three regression analyses are dedicated to video gaming (Section “Videogame Play Increase”). Section “Gender-Related Potentially Addictive Behaviors and Gender-Related Emotions” discusses the combination of results of the previous three subsections to test Hypothesis 4.

### Eating Loss of Control

“Eating loss of control” is a binary dependent variable [from the questionnaire in Supplementary Appendix B): “I feel like I am losing control of my usual diet,” with a “Yes” (1) or “No” (0) answer]. Thus, a logit regression model is used to study the effect of gender (Hypothesis 2) and emotional states (Hypothesis 3) on food overeating. Table 3 provides the estimation results for the logit regression. We interpret the odds ratios of statistically significant variables only. For all these dummy variables, odds ratios greater (resp., smaller) than 1 indicate that those who responded “1” are more (resp., less) likely to lose control over food consumption.

**TABLE 3:** Logistic regression on eating loss of control.

	**Odds ratios**	**Std. Err.**
Female	1.571**	(0.315)
Age 2	1.375	(0.286)
Age 3–4	0.735	(0.276)
Health – 1	0.910	(0.183)
Health – 2	0.919	(0.568)
Lockdown period 1	1.449	(0.400)
Lockdown period 2	1.812	(0.688)
Employed	0.919	(0.197)
Stay at home	0.560**	(0.133)
In relationship	0.659**	(0.132)
Children	0.992	(0.189)
Physical activity 1	1.266	(0.237)
Physical activity 2	0.850	(0.188)
Boredom food	2.012***	(0.408)
Emptiness food	1.813***	(0.380)
Stress food	2.449***	(0.488)
Sadness	2.275***	(0.458)
Discouragement	1.669***	(0.301)
Nervousness	1.085	(0.194)
Cons	0.076***	(0.033)
Observations	1,065	
LR chi^2^ (15)	244.65	
Prob > chi^2^	0.0000	
Pseudo R^2^	0.1994	

Odds ratios are reported: *p < 0.1, **p < 0.05, and ***p < 0.01.

As for gender, the odds of losing control over food if a person was identified as female was 1.571 times more than of males: women were more likely to lose control overeating than men. Furthermore, the former ones were more likely to develop this potentially addictive behavior if they were working outside the home during the lockdown (odds ratio of “Stay at home”: 0.560) and/or if they were not living with a partner during the lockdown (odds ratio of “Living with partner”: 0.659). Therefore, an increase in food consumption was more likely to be developed by women continuing to work outside the home and not having a partner when coming back home after work. All this provides **strong support to Hypothesis 2**.

As for emotional states, all odds ratios of negative emotions are greater than 1, and all but one emotional state variable (nervousness) are significant. Negative emotions which are managed with the help of food consumption all present odds ratios significantly greater than 1: the odds of “Eating loss of control” if a person reported to manage boredom, emptiness, or stress with the help of the food were 2.012, 1.813, and 2.449, respectively, i.e., such a person was more likely to increase food consumption during the lockdown compared to the one who did not report to manage emotions in this way. Furthermore, respondents being sad most of the time (odds ratio 2.275) and/or having a feeling of discouragement about the future (odds ratio 1.669) were more likely to have overeating problems. All this provides **strong support to Hypothesis 3**.

### Smartphone Usage Increase

As in the previous model, our dependent variable – “Smartphone usage increase” – is binary (from the questionnaire in Supplementary Appendix B): “Do you use your smartphone more?” with a “Yes” (1) or “No” (0) answer). Results of the logistic regression are presented in Table 4. Again, we interpret the odds ratios of statistically significant variables only.

**TABLE 4:** Logistic regression on smartphone usage increase.

	**Odds ratios**	**Std. Err.**
Female	2.253***	(0.538)
Age 2	0.688	(0.184)
Age 3–4	0.857	(0.350)
Health – 1	1.628*	(0.481)
Health – 2	1.340	(0.929)
Lockdown period 1	1.358	(0.465)
Lockdown period 2	1.347	(0.658)
Employed	1.065	(0.286)
Stay at home	1.241	(0.385)
In relationship	0.959	(0.256)
Children	0.878	(0.226)
Physical activity 1	0.852	(0.223)
Physical activity 2	1.065	(0.305)
Boredom web	2.320***	(0.639)
Emptiness web	1.166	(0.325)
Stress web	1.783**	(0.500)
Sadness	0.987	(0.342)
Discouragement	1.186	(0.328)
Nervousness	1.814**	(0.463)
Smartphone sms-calls	0.346**	(0.145)
Smartphone soc-network	3.849***	(1.094)
Smartphone games	1.514*	(0.374)
Cons	0.802	(0.554)
Observations	1,056	
LR chi^2^ (18)	123.46	
Prob > chi^2^	0.0000	
Pseudo R^2^	0.1670	

Odds ratios are reported: *p < 0.1, **p < 0.05, and ***p < 0.01.

As for gender, the odds of smartphone usage increase for female respondents are 2.253 times higher than for male respondents: women were more likely to increase smartphone usage than men during the lockdown compared to pre-lockdown levels. Furthermore, the former ones were more likely (resp., less likely) to develop this addictive behavior if the smartphone was used for web connection in social networks (resp., the traditional role of sending SMS and making calls). The odds ratio for “Smartphone SMS-calls” is smaller than 1 (0.346), which means that people who used smartphones mainly for phone calls and SMS were less likely to enhance their smartphone dependence. At the same time, the odds of the use of smartphones increase if a person uses a smartphone for social networks were 3.849 times more than those of the ones who did not. All this provides **strong support to Hypothesis 2**.

Given the significant positive impact of web connection to social networks on smartphone usage increase, when looking at the role of negative emotions that are managed with non-creative activities, we consider those that are managed with connection to the web. We find that boredom, stress, and emptiness managed with connection to the web all have odds ratios greater than 1. However, the positive impact on the increase of smartphone usage is significant for boredom (odds ratio 2.320) and stress (odds ratio 1.783), but not for emptiness. Furthermore, among the three negative emotions elicited independently from a specific non-creative activity – sadness, discouragement, and nervousness –, only the latter has an odds ratio significantly greater than 1 (1.814). Hence, respondents feeling nervous and restless more easily than usual were more likely to increase smartphone usage as compared to pre-lockdown levels, while this was not true for those being sad most of the time and/or having a feeling of discouragement about the future. With this, we can state that **Hypothesis 3 is only partially confirmed**.

### Videogame Play Increase

Here, we rely on the categorical variable “Videogame play increase” (from the questionnaire in Supplementary Appendix B), where respondents have been asked to indicate one out of no activity (“I do not play,” 0), decreased activity (“I spend less time,” 1), stable activity (“My usage is stable, 2”), or increased activity (“I spend more time,” 3) as for videogame play during the lockdown, in comparison to the pre-lockdown activity (see Supplementary Appendix Table A1).

To begin the analysis, we transform the categorical variable “Videogame play increase” of Table 1 and Supplementary Appendix Table A1 into a dummy variable which only accounts for increased vs. non-increased videogame play due to the lockdown: value 1 for increased activity and value 0 for decreased, stable or no activity during the lockdown. With this, in Table 5, we run for “Videogame Play Increase” dummy the same logit regression analysis as for the other two potentially addictive behaviors assessed as behavioral increases with respect to the pre-lockdown levels (Sections “Eating Loss of Control” and “Smartphone Usage Increase”).

**TABLE 5:** Logistic regression on videogame play increase.

	**Coefficient**	**Std. Err.**
Female	–0.358	(0.229)
Age 2	−0.585**	(0.279)
Age 3–4	−0.784*	(0.440)
Health 1	–0.064	(0.251)
Health 2	–0.051	(0.731)
Lockdown period 1	–0.021	(0.330)
Lockdown period 2	0.282	(0.448)
Employed	–0.203	(0.255)
Stay at home	0.046	(0.296)
In relationship	0.130	(0.249)
Children	−0.463*	(0.244)
Physical activity 1	–0.238	(0.234)
Physical activity 2	0.008	(0.261)
Boredom videogames	2.631***	(0.243)
Emptiness videogames	1.040***	(0.283)
Stress videogames	0.386	(0.298)
Sadness	0.167	(0.272)
Discouragement	0.539**	(0.229)
Nervousness	–0.009	(0.221)
Cons	–2.023	(0.523)
Observations	1,064	
LR chi^2^ (15)	567.23	
Prob > chi^2^	0.0000	
Pseudo R^2^	0.4418	

Coefficients are reported: *p < 0.1, **p < 0.05, and ***p < 0.01.

This time for perception convenience Table 5 presents coefficients rather than odds ratios. This is made to allow a clean comparison between this model and the multinomial logistic regression models in Tables 6A,B, which account for the categorical nature of the original variable “Videogame play increase.” Coefficients can be interpreted in the following way: for each predictor, the regression slope is the predicted change in the log odds of falling into the group with increased videogame activity (as compared to the reference group of non-increased activity) per one unit increase on the predictor, holding all other predictors constant.

**TABLE 6A:** Multinomial logistic regression on videogame play increase: category “no activity”.

	**Coefficient**	**Std. Err.**

**Baseline category “stable activity”**		
Female	0.555**	(0.247)
Age 2	–0.307	(0.249)
Age 3–4	−0.654*	(0.350)
Health – 1	–0.037	(0.248)
Health – 2	0.630	(0.784)
Lockdown period 1	0.522*	(0.314)
Lockdown period 2	0.599	(0.478)
Employed	0.144	(0.260)
Stay at home	0.313	(0.291)
In relationship	–0.225	(0.255)
Children	0.034	(0.237)
Physical activity 1	–0.003	(0.245)
Physical activity 2	–0.141	(0.264)
Boredom videogames	−2.884***	(0.404)
Emptiness videogames	−2.257***	(0.564)
Stress videogames	−2.355***	(0.562)
Sadness	–0.104	(0.293)
Discouragement	0.162	(0.246)
Nervousness	0.536**	(0.232)
Cons	0.744	(0.510)
Observations	1,064	
LR chi^2^ (45)	825.03	
Prob > chi^2^	0.0000	
Pseudo R^2^	0.3922	

Coefficients are reported: *p < 0.1, **p < 0.05, and ***p < 0.01.

**TABLE 6B:** Multinomial logistic regression on videogame play increase: category “increased activity”.

	**Coefficient**	**Std. Err.**

**Baseline category “stable activity”**		
Female	–0.154	(0.238)
Age 2	−0.706**	(0.300)
Age 3–4	−1.107**	(0.457)
Health – 1	–0.061	(0.271)
Health – 2	0.095	(0.850)
Lockdown period 1	0.176	(0.342)
Lockdown period 2	0.493	(0.492)
Employed	–0.193	(0.280)
Stay at home	0.108	(0.324)
In relationship	0.078	(0.276)
Children	–0.429	(0.268)
Physical activity 1	–0.254	(0.256)
Physical activity 2	–0.044	(0.290)
Boredom videogames	1.312***	(0.270)
Emptiness videogames	0.558*	(0.295)
Stress videogames	0.094	(0.292)
Sadness	0.142	(0.313)
Discouragement	0.591**	(0.257)
Nervousness	0.218	(0.245)
Cons	–0.523	(0.566)
Observations	1,064	
LR chi^2^ (45)	841.92	
Prob > chi^2^	0.0000	
Pseudo R^2^	0.3801	

*Coefficients are reported: ^∗^*p* < 0.1, ^∗∗^*p* < 0.05, and ^∗∗∗^*p* < 0.01.*

Results in Table 5 show that the coefficient for the female gender is – as predicted – negative, although not significant (*p*-value = 0.119). This suggests that increased videogame play during the lockdown was negatively related to female gender, but not significantly so. It was instead significantly negatively related to age: the increase in videogame play during the lockdown was significantly higher at lower age levels (less than 35 years old). Moreover, according to the negative and significant coefficient of the “Children” variable, respondents who were at home with their children were less likely to increase videogame play, eventually because they (had to) use their time to take care of their children (see, e.g., Shockley et al., 2020).

Finally, all coefficients of videogame-conditional emotional states – boredom, emptiness, and stress managed by videogame play – are positive, but only those of boredom and emptiness are significant, i.e., people who tended to manage these two negative emotions by the means of videogames were more likely to incur into potentially addictive videogame behavior. Finally, feeling discouraged significantly increased the likelihood of playing videogames more.

The multinomial logit regressions of Tables 6A,B shed further light on why no significant gender difference in favor of males is found as for videogame play increase due to the lockdown. Here we rely on the original four values of the categorical variable ‘‘Videogame play increase,’’ which disentangles subjects who did not play at all videogames during the lockdown -- and who were supposedly not playing them also before -- from subjects who did not increase their videogame activity during the lockdown. In the latter category, due to their negligible share, we also include subjects who decreased their videogame activity during the lockdown.^13^ The “stable or decreased activity” category (values 1 and 2 pooled, from now on, “stable activity”) is used as baseline category for both “no play” category “0” and for “increased activity” category “3.” With this, the regression results of Table 6A (resp., Table 6B) indicate which of the independent variables significantly predict whether a person falls into the “no play” video gaming activity category (resp., increased activity) vs. a baseline category which includes stable activity. In other words, the regression results of Table 6A identify the fixed effect of stable playing (baseline) vs. not playing videogames (comparison group) regardless of the lockdown. The regression results of Table 6B identify the increase in videogame play (comparison group) due to the lockdown. As in Table 5, each regression slope in Tables 6A,B is interpreted as the predicted change in log odds of belonging to the comparison group (relative to the baseline group) per unit increase on the predictor.

For the category “0 – no videogame activity,” the coefficient of the Female dummy in Table 6A is positive and significant at the 5% level, hence indicating that being a female rather than a male makes more unlikely to play videogames. This represents a fixed effect of males playing videogames more than females regardless of the lockdown, a further confirmation that videogame play is a male-related non-creative activity.

Moreover, all coefficients of videogame-conditional emotional states – boredom, emptiness, and stress managed by videogame play – are negative and significant, i.e., playing rather than not playing videogames is driven by negative emotions managed through this non-creative activity. An effect of opposite sign is found for nervousness: the log-odds of a case falling into the “no play” category (relative to the “stable” category) is predicted to increase by 0.536 units. Feeling discouraged had no significant impact on videogame play.

For the category “3 – increased videogame activity,” the coefficient of the Female dummy in Table 6B is – as predicted – negative, but far from being significant, hence confirming the results of Table 5: being a female rather than a male does not make more unlikely to increase videogame play during the lockdown. This is clear by comparing the coefficient of the Female dummy in Table 5 and Table 6B (−0.358 and −0.154): the higher absolute value of the coefficient in Table 5 is due to the fixed effect of males playing videogames more than females regardless of the lockdown detected in Table 6A. Therefore, although videogame play was a male-related activity during the lockdown (Table 6A), the gender gap supposedly remained at the pre-lockdown existing levels since the increase in videogame play during the lockdown was unrelated to the player’s gender (Table 6B).

As for age, the effect detected in Table 5 is confirmed: compared to stable users only, the increase in videogame play during the lockdown was significantly higher at lower age levels. Table 6A also confirms the results in Table 5 on the significant positive effect for only a few of the negative emotions, i.e., for only two out of the three videogame-conditional emotional states, namely boredom and emptiness, and, among the unconditional ones, only for discouragement.

With this, we can conclude that the model in Table 5 and the models in Tables 6A,B **do not support Hypothesis 2, while they partially support Hypothesis 3**. As for Hypothesis 2, the significant gender difference detected in Table 6A confirmed that video gaming is a men-related non-creative activity, but the insignificant gender difference found in Tables 5, 6B proved that the lockdown did not bring a gender-related increase in such activity. As for Hypothesis 3, among emotional states, only boredom, emptiness and discouragement had a significant impact in increasing videogame play, although discouragement had no significant impact on stable vs. no videogame activity (Table 6A). The negative impact of nervousness on stable vs. no videogame activity can be seen as confirmation that the most relevant emotion that is managed by videogame activity is boredom, which usually characterizes subjects who are more relaxed and calmer (Walters et al., 1982).^14^

### Gender-Related Potentially Addictive Behaviors and Gender-Related Emotions

We conclude the data analysis with the test of Hypothesis 4, i.e., of whether the negative emotions driving the increase of gender-related non-creative activities are themselves gender-related. Recall that as far as emotional states variables are concerned, our questionnaire includes two types of items: emotions elicited without relating them to specific behavior (unconditional: sadness, discouragement, and nervousness) and emotions elicited conditionally to the (non-creative) activity aimed at managing them (conditional: boredom, emptiness, and stress). Relying on Table 2, at the beginning of section “Results” we have highlighted that gender differences are systematically detected for each of the six emotional states variables, with women showing significantly higher sensitivity than men to unconditional emotions sadness, discouragement, and nervousness, and men showing significantly higher sensitivity to boredom, emptiness, and stress on average over the three non-creative activities they are aimed at managing. Summarizing the results of Tables 3–5, 6A,B, 7 reports the signs of the significant impacts of each of the six negative emotions as for the three potentially addictive behaviors (eating loss of control, smartphone usage increase, and videogame play increase) separately, disentangling by female-related (unconditional) vs. male-related (conditional) emotions and by female-related (food and smartphone) vs. male-related non-creative activities (videogames).

**TABLE 7:** Impact of negative emotions on potentially addictive behaviors, disentangled by gender-related emotion, and gender-related non-creative activity.

		Female-related		Male-related
		Food	Smartphone	Videogames
**Unconditional (female-related)**	Sadness	+	n.s.	n.s.
	Discouragement	+	n.s.	n.s.
	Nervousness	n.s.	+	n.s.
**Conditional (male-related)**	Boredom	+	+	+
	Emptiness	+	n.s.	+
	Stress	+	+	n.s.

*Impacts: “+” significantly positive, and “n.s.” not significant.*

Let us first look at the three female-related emotions (sadness, discouragement, and nervousness), i.e., the first three lines of Table 7. A similar picture emerges: each of them has a significant positive impact (+ in Table 7) on the increase of one of the two female-related non-creative activities (food or smartphone) and a non-significant impact (n.s. in Table 7) on the increase of the male-related non-creative activity (videogames). Spearman’s tests of rank correlation between specific potentially addictive behaviors and specific negative emotions confirm a significant positive correlation between each female-related negative emotions and the increase in each of the two female-related non-creative activities: for eating loss of control, *p*-value < 0.000 for any among sadness, discouragement and nervousness, with lowest Spearman’s rho = 0.16; for smartphone usage increase, *p*-value = 0.010 for sadness, 0.003 for discouragement, and <0.000 for nervousness, with lowest Spearman’s rho = 0.08. A non-significant correlation is found as for the increase in the male-related non-creative activity (videogame): *p*-value = 0.378 for sadness, 0.098 for discouragement, and 0.133 for nervousness, with highest Spearman’s rho = 0.05.

Looking at the three male-related emotions, i.e., the last three rows of Table 7, a different picture emerges: each of these emotions has a significant positive impact on both male-related and female-related non-creative activities. In confirmation of that, a significant positive correlation is detected not only between male-related negative emotions and videogame increase (*p*-value < 0.000 for any among boredom, emptiness, and stress managed by videogame, lowest Spearman’s rho = 0.54), but also between male-related negative emotions and the increase in female-related non-creative activities (for eating loss of control, it is *p*-value < 0.000 for any among boredom, emptiness, and stress managed by food, with lowest Spearman’s rho = 0.32; for smartphone usage increase, it is *p*-value < 0.000 for any among boredom, emptiness, and stress managed by web, with lowest Spearman’s rho = 0.17).

With this, we conclude that **Hypothesis 4 is verified for women but not for men:** the negative emotions driving the increase of female-related non-creative activities are themselves female-related, while the negative emotions driving the increase of male-related non-creative activities are shared by women too.

## Discussion and Conclusion

The sudden outbreak of COVID-19 and the consequent enforcement of the lockdown have abruptly disrupted people’s routines and increased social isolation and financial stress around the world. The psychological impact of this traumatic experience will have short as well as long-run effects. Philosophers and psychologists (e.g., Thomson, 2018) suggest that the presence of trauma, as well as negative states, such as anxiety and depression, may enhance creativity. In a study comparable with ours (general population in France) run during the same period (1st weeks of the 2020 lockdown), Mercier et al. (2021) report that lockdown, despite the negative outcomes that came out of it, may have fostered creativity due to uncertainty and solitude. However, the influence of negative mood on cognitive creativity and emotional creativity remains elusive (Ying et al., 2020). Failure in engaging in a creative activity to overcome uncertainty and solitude might explain people’s increase in everyday non-creative activities, thereby leading to potentially addictive behaviors. Indeed, we find that 26% of our sample felt like losing control of their usual diet; 88% started to use the smartphone more than in the lockdown period, while 64% of those playing videogames reported increased activity with respect to the pre-lockdown period. This significant increase in non-creative activities occurred after only 2 weeks from the beginning of the first lockdown in France (end of March 2020). While Dubey et al. (2020) find an increase in both new and relapse addictive behaviors during the same period, more interestingly, our study reports that, rather than moving to “new” addictions, people in lockdown stuck to their pre-lockdown habits, by investing the additional free time into the same non-creative activities they were “specialized” before the lockdown.

In our study, we focus on food consumption, smartphone usage, and videogame play, and we find a significant gender effect in the increase of these non-creative activities, with gender differences in favor of women (resp., men) being reported for the first two non-creative activities (resp., the last one) by the literature in psychology before COVID-19 (see, e.g., Davis, 2013 for food, Hong et al., 2012 for smartphones, and King et al., 2012 for videogames). Indeed, in our sample women were about 1.6 times more likely than men to losing control of their usual diet, about 2.3 times more likely than men to use smartphones more, and they showed the same propensity as men to play videogames more with respect to the pre-lockdown activity.

As far as the increase in the usage of smartphones, the significant gender effect detected deserves a more thorough discussion. It was reasonable to expect that in the situation of limited freedom of movement people were more likely to rely on technologies to communicate. Thus, consistently with this prediction, our results indicate that during the period of lockdown people mainly used smartphones for communication. However, what is interesting is that women were more likely (resp., less likely) to develop potential smartphone addiction if they used the smartphone for web connection to social networks (resp., for the traditional role of sending SMS and making calls). This result provides insight into gender differences in the dependence on the social networks or in expressing social needs.

Gender effects were also present with respect to the use of these three non-creative activities to manage negative emotions, and to the sensitivity to these emotions. We find that during the 1st weeks of the COVID-19 lockdown in France, a considerable proportion of women and men in our sample experienced negative emotions like sadness (resp., 24 and 18%), discouragement (resp., 40 and 28%), and nervousness (resp., 49 and 32%), with women reporting a significantly higher sensitivity to these negative emotions than the one detected for men. This finding is in line with what other studies have reported for the COVID-19 pandemic affecting women more than it has affected men in terms of the psychological impact of confinement (see, e.g., Guadagni et al., 2020; Ausín et al., 2021). However, when we turn to negative emotions managed through the three non-creative activities mentioned above, we find that men show significantly higher sensitivity than women to boredom, emptiness, and stress, on average over the three non-creative activities they are aimed at managing (61 vs. 48% for boredom, 49 vs. 40% for emptiness, and 39 vs. 32% for stress). The different prevalence of experienced emotions (sadness, discouragement, and nervousness vs. boredom, emptiness, and stress) has a different impact on the increase of non-creative activities. In line with Scitovsky’s (1999) suggested link between boredom and potentially addictive behavior, boredom was detected to be the modal negative emotion that each of the three non-creative activities under scrutiny aimed at mitigating during the first 2 weeks of 2020 lockdown. Interestingly, a strong emotion-behavior specific link was found between boredom and web activity, with the fraction of respondents managing boredom with a web connection (79%) being significantly higher than the one of those managing boredom with food (43%) or videogame (30%) consumption.

To summarize, our empirical results suggest that during the COVID-19 lockdown the tendency to overcome negative emotions with the help of activity that did not require special or creative skills increased the probability for a person to become addicted to this activity. We found that women used food and the web significantly more than men as a remedy for negative emotional states, thereby developing two harmful behavioral patterns, which both usually lead to an increase in the obesity risk. These results proved to be significant, suggesting that negative emotional states, though to a different extend, can serve as triggers for developing potentially addictive behavior when and if not directed to creative actions. However, one has to keep in mind that differences in eating disorders might also be due to hormonal factors (Beydoun, 2014), while differences in evaluating negative emotions as discouragement, sadness, and nervousness might be due to different abilities to express emotions caused by different sensitivities or more realistically to stereotypes and social expectations (Shields et al., 2006).

Recall that we detected these behavioral patterns and their specific emotional drivers by using data from the 1st 2 weeks of the lockdown. We expect the detected behavioral patterns and the links with negative emotions to have become even stronger during the following weeks of the European lockdown in 2020. This is indirectly confirmed by Sabater-Grande et al. (2021), who detected an average lower levels of daily life satisfaction by females in Spain during spring 2020, although females in their study exhibited a stronger tendency to report higher levels of life satisfaction the longer their lockdown forecast.

Our findings confirm those of other recent studies (e.g., Zacher and Rudolph, 2020) that the COVID-19 pandemic represents not only a major medical and economic crisis, but also has a psychological dimension, as it can be associated with declines in key facets of people’s subjective wellbeing. Again, in line with recent studies (e.g., Yan et al., 2021), we report sex differences in emotional reactions and behavioral responses to COVID-19 and related threats. We add to this picture specific findings on gender effects in potentially addictive behaviors and in the negative emotions these behaviors aim at managing. In particular, we show that the negative emotions driving the increase of female-related non-creative activities were themselves female-related, while the negative emotions driving the increase of male-related non-creative activities were shared by women too. This explains the absence of gender differences in the increase of male-related non-creative activities during the lockdown.

As for policy implications, our study suggests once more that designing intervention strategies that account for gender differences in emotional and behavioral responses in facing the COVID-19 pandemic is crucial for these strategies to be effective in the long term. Several other studies have reported evidence implying the need for gender-based public health policies and communication on COVID-19 (see, e.g., Galasso et al., 2020). As for the specific case of potential behavioral addictions, our study suggests that remedies such as sin taxes, legal restrictions, antidepressants, and so on, will be just a symptomatic treatment since the root of the problem is much deeper. Structural reforms in education and economic systems are needed, to help people develop their creative skills and intrinsic motivation for creative behavior, which could prevent us from falling into the vicious circle of the increase of non-creative activities during future lockdown periods that, after COVID-19 outbreaks, do not seem to be so unlikely.

In this regard, an important limitation of our study is worth discussing. The role of developing creative skills as a remedy against the problem of potentially addictive behaviors during the lockdown was not detected as our survey did not contain relevant questions on this issue. This can be the subject for further research on gender differences in COVID-19 side-effects on behavioral addictions.

Another limitation of our study is the unrepresentativeness of our sample as for gender (around 75% of our respondents were female). In the section “Methodology” we hypothesized that the higher online survey participation by females was due to their higher willingness to disclose their psychological health during the lockdown, because of a worse status as compared to males’ one (as our analysis of the section “Results” shows). We leave the test of this explanation of gender unbalance for further research.

Finally, we plan to replicate this study in the future to detect whether our results on gender differences in the impact of COVID-19 lockdown will last after the end of the pandemic, to check whether the detected female-related increases in non-creative activities have become in the meanwhile addictive behaviors.

## Data Availability Statement

The raw data supporting the conclusions of this article will be made available by the authors, without undue reservation.

## Ethics Statement

Ethical review and approval was not required for the study on human participants in accordance with the local legislation and institutional requirements. Written informed consent for participation was not required for this study in accordance with the national legislation and the institutional requirements.

## Author Contributions

FC had the original idea of the survey. FC and CB contributed to the design and distribution of the survey. GA built the review of the literature and was the main coordinator of the data analysis and the writing of the manuscript. GA and AM were involved in the writing of the final version of the manuscript. TS made the first data analysis under the supervision of AM and built the tables and was involved in the writing of the initial version of the manuscript. All authors contributed to the article and approved the submitted version.

## Conflict of Interest

The authors declare that the research was conducted in the absence of any commercial or financial relationships that could be construed as a potential conflict of interest.

## Publisher’s Note

All claims expressed in this article are solely those of the authors and do not necessarily represent those of their affiliated organizations, or those of the publisher, the editors and the reviewers. Any product that may be evaluated in this article, or claim that may be made by its manufacturer, is not guaranteed or endorsed by the publisher.

## Footnotes

1 Source: France Télévisions, LCI, Population, March 28, 2020: www.lci.fr/population/confinement-et-coronavirus-les-violences-conjugales-en-hausse-de-plus-de-30-l-interieur-propose-de-donner-l-alerte-dans-des-pharmacies-2149240.html.

2 As for, e.g., Italy, see: Più Europa, April 22, 2020: www.piueuropa.eu/2020/04/22/COVID-19-violenza-domestica-e-risposta-internazionale.

3 The term “addiction” itself is not always used. For instance, World Health Organization in the 1960s substituted it with the word “dependence” (at least for the case of substance abuse), which is more applicable for various degrees of severity. However, it is still quite common for both professionals and the public to apply the term “addiction.” This paper will consider and use both terms as synonyms.

4 “Novelty,” as opposed to comfort, refers to a source of pleasure that comes from resolving the challenge. The more challenging is the problem the more pleasant and enjoyable is the stimulating effect of the novelty, provided it remains resolvable for a person. The evidence from psychological experiments suggests that novelty has a more powerful stimulating effect when it is accompanied by surprise, conflict, incongruity, and divergence between what is expected and what is experienced. From the point of view of motivation, novelty can be seen as a “desire to know the unknown.” Creative activities are referred to as novelty. Novelty also implies uncertainty, as the resolution of the problem cannot be known in advance. For novelty it is the acquisition of related knowledge and experience that enable us to enjoy it, i.e., “the consumption of novelty is a skilled consumption” (Scitovsky, 1992: 11, Ch.3).

5 As for food, we acknowledge that food preparation and food consumption “rituals,” when people are forced to spend most of their time in the house (under lockdown), could be taken into account as “creative” food-related activities. However, although these food-related activities potentially function as a creative activity to relieve stress, negative effects of COVID-19-induced stress on planning, selecting, and preparing foods can also be expected (see De Backer et al., 2020). Nonetheless, we highlight that the analysis of these opposite effects is out of the scope of this article: the focus of our research is on the mere non-creative part of food consumption (overeating, i.e., losing control of one’s own usual diet).

6 Source Statcounter Global Stats (see https://gs.statcounter.com/social-media-stats/all/france): Twitter had the second-highest market share with 13.84% only.

7 The original questionnaire is in French. In Supplementary Appendix B, we report the English translation. The French version is available from the authors upon request.

8 52.29 females vs. 47.71% males: source INSEE – National Institute of Statistics and Economic Studies (see https://www.insee.fr/fr/statistiques/2381474).

9 51.15 females vs. 48.85% males: source NapoleonCat (see https://napoleoncat.com/stats/facebook-users-in-france/2020/03/).

10 Starting from official data from INSEE in the period of our survey (https://www.insee.fr/fr/statistiques/2381474) and restricting the official statistics to French citizens aged more than 18, we find an age distribution more skewed to the right (28.41% aged 18–35, 31.04% aged 36–55, 28.99% aged 56–75, 11.56% aged more than 75) than ours (respectively: 64.49, 27.87, 7.27, and 0.37%).

11 Weighting the age distribution of French population in 2020 by the age distribution of Facebook users in France in the same period of our survey (March 2020: see https://napoleoncat.com/stats/facebook-users-in-france/2020/03/), Facebook French users’ age distribution becomes 46.14% aged 18–35, 35.18% aged 36–55, 14.51% aged 56–75, 4.18% aged more than 75. This distribution is very close to the age distribution of our sample (respectively: 64.49, 27.87, 7.27, and 0.37%), which mirrors the rank of age categories of social media users in France.

12 Source: SELL (Syndicat des Editeurs de Logiciels de Loisirs), “L’Essentiel du Jeu Vidéo: Les Français et le Jeu Vidéo” (https://www.sell.fr/sites/default/files/essentiel-jeu-video/ejv_novembre_2020_def_web-compresse_0.pdf).

13 Since subjects with decreased videogame activity only represent 1.57% of the sample (17 out of 1,083 responses to this question), results of the multinomial logit regression in Tables 6, 7 are unchanged if we consider these subjects as a separate category, like in the original version of the “Videogame play increase” variable of Table 1 and Supplementary Appendix Table A1. Results of the multinomial logit regression with four separate categories are available upon request to the authors.

14 This is in line with the theory of psychological reversal (Apter, 1984, 2001), where boredom and relaxation are categorized as emotions with similarly low arousal and opposite hedonic tone, respectively unpleasant and pleasant.
