# Supplementary material for: Gender Differences in the Impact of COVID-19 Lockdown on Potentially Addictive Behaviors: An Emotion-Mediated Analysis
Source: Front Psychol. 2021 Nov 15;12:703897. doi: 10.3389/fpsyg.2021.703897 (PMC8634025; doi:10.3389/fpsyg.2021.703897)
Supplement: Supplementary file 1 [file Data_Sheet_1.pdf]

APPENDIX

APPENDIX A

TABLE A1 | Description of selected variables.

Variables	Description
**Socio-demographics**	
Female	Dummy = 1 if respondent is female
Age	1 if 18-35; 2 if 36-55; 3 if 56-75; 4 if > 75 years old
Health	0: good health condition; –1: good general condition with one or more chronic pathologies; –2: one or more serious chronic pathologies
Lockdown period	0: less than 1 week; 1: between 1 and 2 weeks; 2: between 2 and 3 weeks
Employed	Dummy = 1 if respondent is employed (daily commuting or teleworking)
Stay at home	Dummy = 1 if respondent stays at home (not employed, or teleworking)
In relationship	Dummy = 1 if respondent is in relationship
Children	Dummy = 1 if respondent has children
Physical activity	0: none; 1: less than 30 min per day and less than 5 times per week (infrequent); 2: at least 30 min per day and at least 5 times a week (frequent)
**Emotional states**	
Boredom food	Dummy = 1 if boredom is managed with food
Boredom web	Dummy = 1 if boredom is managed with web
Boredom videogame	Dummy = 1 if boredom is managed with videogames
Emptiness food	Dummy = 1 if emptiness is managed with food
Emptiness web	Dummy = 1 if emptiness is managed with web
Emptiness videogame	Dummy = 1 if emptiness is managed with videogames
Stress food	Dummy = 1 if stress is managed with food
Stress web	Dummy = 1 if stress is managed with web
Stress videogame	Dummy = 1 if stress is managed with videogames
Sadness	Dummy = 1 if respondent feels sad most of the time
Discouragement	Dummy = 1 if respondent has a feeling of discouragement about the future
Nervousness	Dummy = 1 if respondent feels nervous and restless more easily than usual
**Non-creative activities**	
Smartphone sms-calls	Dummy = 1 if respondent uses smartphone for calls and sms
Smartphone soc-network	Dummy = 1 if respondent uses smartphone for social networks
Smartphone games	Dummy = 1 if respondent uses smartphone for games
Videogame play	Dummy = 1 if respondent plays videogames
**Potentially addictive behaviors**	
Eating loss of control	Dummy = 1 if respondent feels like losing control of his/her usual diet
Smartphone usage increase	Dummy = 1 if respondent starts to use smartphone more
Videogame play increase	0: no activity; 1: decreased activity; 2: stable activity, 3: increased activity

APPENDIX B

QUESTIONNAIRE

[English Translation from French]

Impact of the lockdown on the general population in the context of the COVID-19 pandemic

This questionnaire, carried out by the Addictology Unit of the Archet Hospital in Nice, is open to any adult person undergoing lockdown. Its aim is to evaluate the impact of the lockdown on the state of psychological health and to assess the consumption of addictive products within the general population and the influence of restrictive measures on them. It will take you less than 10 minutes.

1. Sex
   One answer only
   - Female
   - Male

2. Age
   One answer only
   - 18-35
   - 36-55
   - 56-75
   - 76 or more

3. Concerning your health:
   One answer only
   - I am in good health.
   - I am in good general condition and have one or more chronic diseases.
   - I have one or more serious chronic diseases.

4. You have been in lockdown since:
   One answer only
   - less than one week.
   - between one week and 15 days.
   - between 15 days and 1 month.
   - more than 1 month.

5. Professionally, currently:
   One answer only
   - I am teleworking.
   - I continue to go to work.
   - I do not work.

6. Currently, I live:
   Multiple answers possible
   - alone.
   - with my partner.
   - with one or more children.
   - with another adult who is not my partner.
   - with pet(s).

7. Physical activity takes:
   One answer only
   - less than 30 minutes of my time per day and less than 5 times per week.
   - at least 30 minutes of my time per day and at least 5 times per week.
   - I do not do any physical activity.

8. My sexual activity:
   One answer only
   - o has increased.
   - o has decreased.
   - o is the same.

9. How do you handle boredom?
   Multiple answers possible
   - o With food.
   - o With social networks/the Internet.
   - o With videogames.
   - o With the use of alcohol.
   - o With the use of drugs.
   - o With the use of cocaine.
   - o With the use of cannabis.
   - o Other : ________________________

10. I feel sad most of the time:
    One answer only
    - o Yes
    - o No

11. I feel discouraged about the future:
    One answer only
    - o Yes
    - o No

12. How do you handle the feeling of emptiness?
    Multiple answers possible
    - o With food.
    - o With social networks/the Internet.
    - o With videogames.
    - o With the use of alcohol.
    - o With the use of drugs.
    - o With the use of cocaine.
    - o With the use of cannabis.
    - o Other : ________________________

13. I feel more easily nervous and agitated than usual:
    One answer only
    - o Yes
    - o No

14. How do you handle stress?
    Multiple answers possible
    - o With food.
    - o With social networks/the Internet.
    - o With videogames.
    - o With the use of alcohol.
    - o With the use of drugs.
    - o With the use of cocaine.
    - o With the use of cannabis.
    - o Sport at home and outside.
    - o Other : ________________________

15. About anxiolytic treatments:
    One answer only

- I have been taking an anti-anxiety medication (XANAX, SERESTA, LEXOMIL...) since the lockdown.
- I have increased the dosage of my usual anxiolytic medication since the lockdown.
- I do not take anxiolytic medication.

16. I have more difficulty falling asleep:
    One answer only
    - Yes
    - No

17. I feel that my sleep is of poorer quality (night-time awakenings, shortened sleep time, tiredness on waking...):
    One answer only
    - Yes
    - No

18. About hypnotic treatments (ex : zopiclone, zolpidem, noctamide, théralène, donormyl...)
    One answer only
    - I use hypnotics when I did not need them before. (théralène, imovane, stilnox...).
    - I have increased the dosage of my usual hypnotic treatment.
    - I do not take any hypnotic treatment.

19. I feel like I am losing control of my usual diet:
    One answer only
    - Yes
    - No

20. Tobacco consumption:
    Multiple answers possible
    - I have increased my consumption.
    - I have reduced my consumption.
    - My consumption is the same.
    - I stopped smoking.
    - I do not smoke

21. If I am a smoker, I have more difficulty getting tobacco:
    One answer only
    - Yes
    - No

22. Do you sometimes have a strong urge to use tobacco that is very difficult to control?
    One answer only
    - Yes
    - No

23. Alcohol consumption:
    One answer only
    - I have increased my consumption.
    - I have reduced my consumption.
    - My consumption is the same.
    - I stopped drinking.
    - I do not drink.

24. Did you have to seek medical attention due to acute alcohol abuse:
    One answer only
    - Yes
    - No

25. Have you had to seek medical help for alcohol withdrawal symptoms (tremors, sweating, anxiety, hallucinations, epilepsy, delirium tremens...)
    One answer only
    - Yes
    - No

26. Do you use any other substances:
    One answer only
    - Cannabis
    - Cocaine
    - Heroin
    - No
    - Other: ________________________

27. If I use, I have a harder time than usual to get these substances:
    One answer only
    - Yes
    - No

28. Sometimes, I have a strong urge to use these substances, very difficult to control:
    One answer only
    - Yes
    - No

29. Have you had to seek medical attention because of withdrawal symptoms related to these substances (shaking, sweating, anxiety, internal tension, digestive problems, pain...)
    One answer only
    - Yes
    - No

30. Regarding the use of social networks (snapchat, instagram, twitter, facebook...):
    One answer only
    - I spend more time on it.
    - I spend less time on it.
    - My usage is stable.
    - I do not use these social networks.

31. Regarding videogames:
    One answer only
    - I spend more time on it.
    - I spend less time on it.
    - My usage is stable.
    - I do not play.

32. Do you use your smartphone more?
    One answer only
    - Yes
    - No

33. For what reasons do you use your smartphone?
    Multiple answers possible
    - Calls.
    - Social networks.
    - SMS.
    - Games.
    - Other: ________________________

34. What is your strategy to better cope with the lockdown? (on 2 lines max)
    ____
    ____
